# Rural-urban migration patterns and mental health diagnoses of adolescents and young adults in British Columbia, Canada: a case-control study

**DOI:** 10.1186/1753-2000-4-13

**Published:** 2010-05-13

**Authors:** Stefania Maggi, Aleck Ostry, Kristy Callaghan, Ruth Hershler, Lisa Chen, Amedeo D'Angiulli, Clyde Hertzman

**Affiliations:** 1 Institute of Interdisciplinary Studies and Department of Psychology, Dunton Tower Room 2210, Carleton University, 1125 Colonel By Drive, Ottawa, ON, K1S 5B6, Canada; 2 Department of Geography, University of Victoria, PO BOX 3060 STN CSC, Victoria, BC, V8W 3R4, Canada; 3 Thompson Rivers University, Box 3010, 900 McGill Road, Kamloops, BC, V2C 5N3, Canada; 4 Human Early Learning Program, University of British Columbia, 4th Floor, Library Processing Centre, 2206 East Mall, Vancouver, BC, V6T 1Z3, Canada

## Abstract

**Background:**

The identification of mental health problems early in life can increase the well-being of children and youth. Several studies have reported that youth who experience mental health disorders are also at a greater risk of developing psychopathological conditions later in life, suggesting that the ability of researchers and clinicians to identify mental health problems early in life may help prevent adult psychopathology. Using large-scale administrative data, this study examined whether permanent settlement and within-province migration patterns may be linked to mental health diagnoses among adolescents (15 to 19 years old), young adults (20 to 30 years old), and adults (30 years old and older) who grew up in rural or urban communities or migrated between types of community (N = 8,502).

**Methods:**

We conducted a nested case-control study of the impact of rural compared to urban residence and rural-urban provincial migration patterns on diagnosis of mental health. Conditional logistic regression models were run with the following International Classification of Diseases, 9th Revision (ICD-9) mental health diagnoses as the outcomes: neurotic disorders, personality disorder, acute reaction to stress, adjustment reaction, depression, alcohol dependence, and nondependent drug abuse. Analyses were conducted controlling for paternal mental health and sociodemographic characteristics.

**Results:**

Mental health diagnoses were selectively associated with stability and migration patterns. Specifically, adolescents and young adults who were born in and grew up in the same rural community were at lower risk of being diagnosed with acute reaction to stress (OR = 0.740) and depression (OR = 0.881) compared to their matched controls who were not born in and did not grow up in the same rural community. Furthermore, adolescents and young adults migrating between rural communities were at lower risk of being diagnosed with adjustment reaction (OR = 0.571) than those not migrating between rural communities. No differences were found for diagnoses of neurotic disorders, personality disorder, alcohol dependence, and nondependent drug abuse.

**Conclusions:**

This study provides some compelling evidence of the protective role of rural environments in the development of specific mental health conditions (i.e., depression, adjustment reaction, and acute reaction to stress) among the children of sawmill workers in Western Canada.

## Background

Considerable theoretical debate has focused on the relationships between the development of mental health problems among youth and the role played by environmental stressors such as acute traumatic events, chronic strain and adversity, accumulation of stressful life events, and daily challenges [1-4]. The most notable factors known to have a profound impact on youth mental health include exposure to neighborhood violence [5]; parental chronic illness [6,7], and poverty and economic hardship [8]; as well as parental unemployment, which may add further stress in the form of increased parental alcohol intake, home violence, and child abuse [9].

Much evidence shows that several of these stressors may vary according to where individuals live. That is, the economy and social environment of the communities where youth live may be associated with the degree to which parents are able to find jobs, rely on the necessary networks of social support to cope with challenging times, and provide their children with opportunities for healthy development (for comprehensive reviews, see [10,11]). Since the extent to which these stressors are present may differ between rural versus urban communities, we explore whether exposure to urban or rural environments places youth and young adults at greater risk for poor mental health outcomes.

### Mental health and rurality

Research shows that youth and young adults often struggle with mental health problems such as depression, anxiety, and stress-related conditions. A recent World Health Report estimated that 10%-20% of youth worldwide experience one or more mental health disorders [12]. Several studies have also reported that youth who experience mental health disorders are at greater risk of developing psychopathological conditions later in life (e.g., [13,14]). These results suggest that in addition to increasing the well-being of children and youth, the ability of researchers and clinicians to identify mental health problems early in life may help prevent adult psychopathology.

One of the issues that has stimulated much research on the impact of community-level influences on mental health is whether people living in urban environments are at greater or lesser risk than people living in rural environments. The question may have been motivated by the social construct of the *rural idyll *- a notion that has been consistently influential since the 1960s (see [15-17]) - that is, the underlying discourse that rural areas promote a peaceful and harmonious lifestyle, whereas cities are generally associated with chaos, noise, stress, and challenging living conditions typical of large metropolitan areas [18,19]. Accordingly, one common expectation is that exposure to peaceful rural environments should positively impact people's mental health.

Several studies have investigated whether or not the features of rural communities that tend to evoke images of tranquility - such as beautiful landscapes, privacy from neighbors, and harmony with nature - actually minimize mental health disorders [20-24]. Interestingly, older studies tend to report that urban youth are at higher risk for mental health problems, while more recent studies seem to suggest the opposite. For example, it has been reported that mental health disorders among adolescents from rural communities are increasing to the point of equaling or exceeding those of urban youth [25], especially with respect to drug and alcohol use and abuse [26,27]. Similarly, Gordon and Caltabiano [28] have shown rural-urban differences with regard to self-esteem of adolescents (with rural youth scoring lower than their urban counterparts) and engagement with deviant leisure behaviors such as drug and alcohol use (with rural youth being more likely to engage in such behaviors than urban youth). Despite some results indicating differences in the mental health of youth from rural and urban communities, many other studies have not detected significant differences [19,29-31].

The contradictory results may be partly attributable to the fact that what constitutes "rurality" versus "urbanity" is rarely explicit in studies [17]. In addition, most studies are cross-sectional, focus on a limited number of mental health conditions, or rely on self-report measures. These problems reflect the practical difficulty of considering communities as complex entities and, also, of dealing with the dynamic time component involved in the development of mental health outcomes.

### Mental health and migration patterns

In addition to rurality or urbanity, one important but mostly neglected aspect that can also significantly impact mental health outcomes is the individual history of migration from one place to another, especially when the place of origin differs significantly from the place of arrival. In North American societies, a significant proportion of the population migrate at least once in a lifetime, and many people change community of residence multiple times. Some migrate from urban to rural communities (or vice versa), while others migrate within urban communities or within rural communities only. For instance, census reports for 2006 indicate that approximately 14% of the Canadian population had migrated in the previous year, and 19% had migrated within the previous 5 years [32].

The mobility of populations has been of interest to researchers attempting to uncover the impact of migration patterns on adolescent mental health. Studies have suggested that adolescents who change residence show higher rates of mental disorders. For example, McGee and colleagues [18] found that adolescents who had frequent changes of residence were more likely to have higher rates of mental health diagnoses and higher levels of help-seeking, as well as lower levels of social competence. These lower levels of social competence are thought to be related to difficulties in forming relationships with peers [18].

A study conducted by Mullick and Goodman [31] on 5-10 year olds in Bangladesh found that migrating from rural to urban communities had a negative impact on mental health. Dudley and associates [33] found that youth who migrate from urban to rural areas were more likely to commit suicide than youth migrating from rural to urban areas. Thus, there is a body of evidence that suggests that an individual's mental health can be influenced by migration.

The purpose of the present study is to examine whether, in addition to permanent settlement in urban or rural communities, migration patterns within the province of British Columbia (Canada) may also be linked to mental health diagnoses among adolescents and young adults. We hope to contribute to the limited data and literature about rural mental health among youth, since few studies have investigated the effects of migration in conjunction with permanent settlement. To our knowledge, this is the first Canadian population-based study to investigate mental health diagnoses in adolescents and young adults by exploring the effects of rurality-urbanity and migration patterns through analysis of large-scale administrative data.

## Method

This study is based on a cohort of male sawmill workers (N = 28,794) on whom data was first gathered in the mid-1990s to study the effects of chlorophenol antisapstain exposure among British Columbian sawmill workers [34]. Recently, the original study cohort has been extended to investigate the association between job history, work stress, and health outcomes among the cohort participants and their children [35-37]. For the present study, personnel records for workers who had worked in one of 14 sawmills for at least one year between 1950 and 1998 were accessed and compiled. The birth files from the British Columbia provincial vital statistics registry were used to identify the children of the sawmill workers who were born between 1952 and 2000. Probabilistic linkage techniques were used to identify the study participants and their mental health diagnoses. More specifically, to link records of the children to those of the fathers, we used the Medical Services Plan (MSP) number (the equivalent to a health personal number), gender of the child, date of birth, surname, and given names. This probabilistic technique yielded a success rate of 87%. A total of 37,827 children of sawmill workers were identified, forming an offspring cohort that includes individuals of varying age, ranging from young children to adults.

Mental health information of children of sawmill workers was gathered from the provincial administrative health data. The Canadian health system is public and universally accessible and it is regulated at the provincial level. In British Columbia, individuals experiencing mental health problems can be evaluated by mental health professionals at public hospitals or medical clinics. Every encounter that occurs between patients and health professionals is recorded on administrative forms that are sent and stored at the British Columbia Ministry of Health. The reason for medical visit or hospitalization (which can include a diagnosis if one is provided), and personal health information are recorded on such forms. This individual-level administrative health information is available to researchers who have obtained approval as the result of a stringent process of review of ethical standards and scientific rigor. Such data, which also include codes for mental health diagnoses in accordance with international code systems, are accessed at the British Columbia Linked Health Database (BCLHDB). Ethical approval was obtained from the University of British Columbia (UBC) and the British Columbia Ministry of Health to conduct a series of studies on the health of sawmill workers and their children.

### Study participants

For the children of sawmill workers to be eligible for this study, the fathers must have worked at least one year in one of the study sawmills while their children were between the ages of 0 and 16 years. A total of 19,833 children of sawmill workers satisfied the eligibility criteria for inclusion. Our study focuses specifically on mental health diagnoses that were assigned to children of sawmill workers at different times from early childhood to young adulthood. Therefore, the sample for this study consists of a total of 8,508 participants: 2,127 cases and 6,381 controls (3 matched controls on age and gender for each case). Table 1 describes the sociodemographic characteristics of this sample.

**Table 1:** Sociodemographic Characteristics of Fathers and Children (N = 8,508)

Sociodemographic Characteristics	
Age of Children at Diagnosis	Mean = 27.8 SD = 7.8 Minimum = 14 Maximum = 48

	Frequency (%)

Gender of the Children	
*Females*	2456 (28.9)
*Males*	6052 (71.1)
Age at Diagnosis	
*<20 years of age*	1376 (16.2)
*20-30 years of age*	4232 (49.7)
*>30 years of age*	2900 (34.1)
Marital Status of the Father	
*Married*	7297 (92.1)
*Separated, single, or widowed*	629 (7.9)
Ethnicity of the Father	
*Caucasian*	7332 (86.2)
*Sikh*	955 (11.7)
*Asian or Chinese*	181 (2.1)
Mental Health of the Father	
*Diagnosis before children's diagnosis*	2135 (25.1)
*No diagnosis before children's diagnosis*	6376 (74.9)
Alcoholism of the Father	
*Diagnosis before children's diagnosis*	750 (8.8)
*No diagnosis before children's diagnosis*	7758 (91.2)
Suicidal Behavior of the Father	
*Diagnosis before children's diagnosis*	64 (0.8)
*No diagnosis before children's diagnosis*	8444 (99.2)
Job Level of the Father	
*Manager*	664 (7.8)
*Tradesman*	2718 (31.9)
*Skilled Worker*	1678 (19.7)
*Unskilled Worker*	3448 (40.5)
Urban-Rural Migration of the Children	
*Urban*	1853(22.1)
*Rural*	1780 (21.2)
*Urban migrators*	1215 (14.5)
*Rural to Urban*	1956 (23.3)
*Rural migrators*	1585 (18.9)

### Mental health outcomes

International Classification of Diseases, 9th Revision (ICD-9) criteria and codes for children were used to diagnose mental health problems among individuals between the ages of 15 and 19, whereas adult ICD-9 criteria and codes were used to diagnose mental health problems among individuals 20 years of age and older.

Mental health diagnoses for which there were less than 30 cases were not selected, because the ratio between participants and independent variables would have not been sufficient. The selected diagnoses were neurotic disorders (e.g., anxiety state, obsessive-compulsive disorders, phobic state), personality disorders, acute reaction to stress, adjustment reactions, depression, alcohol dependence, and nondependent drug abuse. Table 2 indicates the number of cases and controls that have been identified for each of the above mental health conditions.

**Table 2:** Number of Cases per IDC-9 Mental Health Diagnosis and Matched Controls (n = 8,218)

IDC-9 codes	Mental Health Diagnosis	Cases	Controls	Total
300	Neurotic Disorders	463	1389	1852
301	Personality Disorder	113	339	452
308	Acute Reaction to Stress	229	705	934
309	Adjustment Reaction	305	915	1220
311	Depression	830	2490	3320
303	Alcohol Dependence	36	108	144
305	Nondependent Drug Abuse	74	222	296

### Rural-Urban Migration Patterns

Statistics Canada offers different definitions of rurality - based on population size, density or proximity to urban centres - and recommends that the selection of specific definitions of rurality be guided by the research question of any given study [38]. In British Columbia there are two large metropolitan centres (Vancouver and Victoria) located in the southern part of the province, and a collection of medium to small towns with low density population distributed across the interior, the northern part of the province, and Vancouver Island. Therefore, we selected a definition of rurality based on population size, whereby communities with fewer than 100,000 people are considered rural and communities with 100,000 people or more are considered urban.

Health information records were inspected for the periods between birth and time at diagnosis to identify migration patterns among the study participants. Definitions of migration patterns were based on changes to the participants' postal codes that were associated with records of health services utilization and provided by the local health authorities. Individuals could have been born in and stayed in rural or urban communities within the province of British Columbia, or moved from rural to urban communities within British Columbia, or vice versa. The following three migration patterns have therefore been identified to describe within province migration: urban to rural (0 = no and 1 = yes); rural to rural (0 = no and 1 = yes); and rural to urban (0 = no and 1 = yes). The following two additional migration patterns were identified to describe participants who had moved away from the province of British Columbia, and for whom we did not have information about the place of destination: urban (0 = stayed and 1 = moved); rural (0 = stayed and 1 = moved).

It is worth noting that an 'urban to urban' migration pattern could not be included in the present study. The original cohort (i.e., the fathers) was indentified among workers of sawmills located in British Columbia in the early 1980s. Urban communities in British Columbia, that is, those with population over 100,000 dwellings, are the cities of Vancouver, Victoria, and Kelowna. Of these, only Vancouver still has a sawmill, while Kelowna's sawmill closed in the late 1980s, and Victoria never had one. Therefore the likelihood of migration for work from an urban sawmill community to another urban sawmill community was largely non-existent among our study cohort.

### Control variables

While the study focuses on the effect of rural-urban migration patterns on mental health of the children's cohort, there are some potential variables that need to be accounted for in the analysis. These variables are the sociodemographic characteristics, the mental health, and the employment history of the fathers.

The following sociodemographic characteristics were obtained from the sawmill employment records: duration of employment (continuous variable); job mobility (classified as upward, downward, or stable); type of employment (one dummy variable for trades, one dummy variable for skilled, and one dummy variable for unskilled; management as referent); ethnicity (one dummy variable for Chinese and one dummy variable for Sikh; Caucasian as referent); and marital status of the fathers (one dummy variable; married as referent). ICD-9 mental health diagnosis (father had been diagnosed with any mental health conditions; one dummy variable; no diagnosis as referent); suicidal behaviors (father had attempted or completed suicide; one dummy variable; no diagnosis as the referent); and alcohol dependence (one dummy variable; no diagnosis as the referent) were obtained from the BCLHDB.

### Analysis

Using survival-time to case-control on STATA 8.0, three controls were selected for each mental health case matched on age and gender. Controls were chosen randomly with replacement from the set at risk. The set at risk were all the offspring of the sawmill worker's cohort, born between 1952 and 2000, whose father had worked in a study sawmill for at least one year during the first 16 years of the child's life. These could be anyone at risk who also satisfied the matching criteria who had not been diagnosed with a mental health condition at the time of diagnosis of the case. Given this procedure, it is possible that a participant is a control in the analysis pertaining to a specific diagnosis, but a case in the analysis pertaining to another diagnosis. For example, a participant may be at risk for depression and be used as a control in such analysis, but also be used as a case for nondependent drug abuse if he/she was assigned such a diagnosis.

Statistical analyses were conducted using conditional logistic regression on STATA 8.0. First, a series of seven univariate analyses (one for each diagnosis) were conducted to identify associations between the five migration patterns (i.e., urban, urban to rural, rural, rural to urban, rural to rural) and mental health outcomes. Second, we conducted a series of four separate multivariate analyses, one for each of the outcomes that yield significant associations with migration patterns. In these analyses we controlled for the following paternal characteristics: duration of employment, paternal ethnicity, marital status, paternal alcohol dependence, mental health of the father, suicidal behavior of the father, and type of employment.

## Results

Results of the univariate analyses are reported in Table 3. Four of the six mental health diagnostic groups had at least one migration category where the 95% confidence interval around the odds ratio excluded 1.0: *nondependent drug abuse*, *acute reaction to stress*, *adjustment reaction*, and *depression*. Multivariate analyses were conducted for these diagnoses, as reported in Table 4.

**Table 3:** Risk of IDC-9 Mental Health Diagnoses Associated with Rural-Urban Migration Patterns

	Odds Ratio	SE	z	95% CI
*Neurotic Disorders (1852)*				
Urban	0.988	0.061	-0.20	0.876-1.11
Urban to Rural	1.04	0.075	0.60	0.907-1.20
Rural	0.923	0.057	-1.29	0.817-1.04
Rural to Urban	1.04	0.621	0.66	0.925-1.17
Rural to Rural	1.08	0.069	1.21	0.953-1.22
				
*Acute Reaction to Stress (934)*				
Urban	1.09	0.084	1.08	0.934-1.26
Urban to Rural	1.10	0.099	1.11	0.927-1.32
Rural	0.756	0.064	3.29	0.64-0.893
Rural to Urban	0.970	0.074	0.40	0.835-1.13
Rural to Rural	1.19	0.093	2.25	1.02-1.39
				
*Depression (3320)*				
Urban	1.07	0.051	1.33	0.970-1.17
Urban to Rural	0.884	0.051	-2.12	0.789-0.99
Rural	0.930	0.045	-1.47	0.845-1.02
Rural to Urban	1.04	0.049	0.81	0.947-1.14
Rural to Rural	1.11	0.054	2.23	1.01-1.23
				
*Personality Disorders (452)*				
Urban	1.02	0.141	0.17	0.782-1.34
Urban to Rural	1.08	0.179	0.46	0.780-1.49
Rural	0.865	0.128	-0.98	0.647-1.16
Rural to Urban	0.995	0.138	-0.03	0.759-1.31
Rural to Rural	1.13	0.169	0.80	0.840-1.51
				
*Adjustment Reaction (1220)*				
Urban	1.05	0.099	0.48	0.869-1.26
Urban to Rural	1.35	0.140	2.89	1.10-1.65
Rural	0.595	0.062	-4.97	0.485-0.73
Rural to Urban	1.15	0.105	1.57	0.965-1.38
Rural to Rural	1.10	0.105	1.01	0.914-1.33
				
*Alcohol Dependence (144)*				
Urban	0.854	0.154	-0.88	0.599-1.22
Urban to Rural	1.06	0.190	0.32	0.745-1.50
Rural	0.889	0.145	-0.72	0.645-1.22
Rural to Urban	1.00	0.155	0.00	0.738-1.35
Rural to Rural	1.26	0.200	1.47	0.926-1.72
				
*Nondependent Drug Abuse (296)*				
Urban	0.754	0.116	-1.83	0.557-1.02
Urban to Rural	1.26	0.199	1.47	0.926-1.72
Rural	0.684	0.102	-2.55	0.510-0.92
Rural to Urban	1.05	0.143	0.34	0.802-1.37
Rural to Rural	1.38	0.201	2.20	1.04-1.83

*p < .05

**Table 4:** Results of the Multivariate Analysis

	Odds Ratio	SE	z	P > |z|	95% CI
*Nondependent Drug Abuse (296)*					
Duration of Employment	0.982	0.016	-1.11	0.265	0.951-1.01
Trades Worker	1.22	0.325	0.73	0.463	0.721-2.05
Skilled Worker	1.13	0.319	0.45	0.655	0.654-1.97
Unskilled Worker	1.54	0.410	1.62	0.105	0.914-2.60
Rural	0.935	0.190	-0.33	0.739	0.627-1.39
Rural to Rural	1.42	0.289	1.72	0.086	0.952-2.11
Chinese	0.187	0.139	-2.25	0.024*	0.043-0.80
Sikh	0.384	0.091	-4.04	0.000**	0.241-0.61
Paternal Mental Health	1.78	0.266	3.87	0.000**	1.33-2.39
Paternal Alcoholism	1.80	0.633	1.68	0.094	0.905-3.58
Paternal Suicidal Behaviors	2.01	1.09	1.30	0.195	0.698-5.80
Marital Status	1.00	0.031	0.11	0.914	0.944-1.07
					
*Acute Reaction to Stress (934)*					
Duration of Employment	0.989	0.008	-1.28	0.202	0.973-1.01
Trades Worker	1.01	0.139	0.07	0.947	0.771-1.32
Skilled Worker	1.37	0.200	2.17	0.030*	1.03-1.83
Unskilled Worker	1.34	0.185	2.10	0.036*	1.02-1.75
Rural	0.740	0.078	-2.86	0.004*	0.603-0.91
Rural to Rural	1.03	0.103	0.32	0.750	0.849-1.26
Chinese	0.314	0.101	-3.60	0.000**	0.167-0.59
Sikh	0.649	0.077	-3.66	0.000**	0.515-0.82
Paternal Mental Health	1.33	0.105	3.65	0.000**	1.14-1.55
Paternal Alcoholism	1.38	0.267	1.64	0.101	0.940-2.01
Paternal Suicidal Behaviors	1.23	0.360	0.72	0.470	0.697-2.19
Marital Status	0.974	0.018	-1.40	0.162	0.938-1.01
					
*Adjustment Reaction (1220)*					
Duration of Employment	0.998	0.010	-0.21	0.834	0.978-1.02
Trades Worker	1.10	0.179	0.59	0.553	0.801-1.52
Skilled Worker	1.59	0.269	2.76	0.006*	1.15-2.22
Unskilled Worker	1.34	0.217	1.81	0.070	0.976-1.84
Rural	1.05	0.134	0.40	0.688	0.814-1.36
Rural to Rural	0.571	0.075	-4.27	0.000**	0.442-0.74
Chinese	0.253	0.112	-3.10	0.002*	0.106-0.60
Sikh	0.606	0.085	-3.55	0.000**	0.460-0.80
Paternal Mental Health	0.604	0.067	-4.52	0.000**	0.485-0.75
Paternal Alcoholism	0.542	0.108	-3.09	0.002*	0.368-0.80
Paternal Suicidal Behaviors	2.64	0.967	2.65	0.008*	1.29-5.41
Marital Status	1.04	0.022	1.63	0.104	0.993-1.08
					
*Depression (3320)*					
Duration of Employment	0.983	0.005	-3.33	0.001*	0.973-0.99
Trades Worker	1.07	0.087	0.83	0.407	0.912-1.25
Skilled Worker	1.22	0.104	2.30	0.021*	1.03-1.44
Unskilled Worker	1.16	0.095	1.83	0.068	0.989-1.36
Rural	0.806	0.057	-3.06	0.002*	0.702-0.93
Rural to Rural	0.980	0.062	-0.32	0.753	0.867-1.11
Chinese	0.348	0.068	-5.37	0.000**	0.237-0.51
Sikh	0.743	0.051	-4.36	0.000**	0.650-0.85
Paternal Mental Health	1.28	0.064	4.98	0.000**	1.16-1.41
Paternal Alcoholism	1.69	0.216	4.06	0.000**	1.31-2.17
Paternal Suicidal Behaviors	1.16	0.235	0.71	0.478	0.776-1.72
Marital Status	0.989	0.011	-1.02	0.308	0.968-1.01

*p < .05

**p < .01

Odds ratio (OR) analyses revealed that after controlling for important paternal characteristics, rural stability is significantly associated with *acute reaction to stress *and *depression*. Specifically, individuals who were born in and grew up in the same rural community were approximately 25% less likely to be diagnosed with *acute reaction to stress *(OR = 0.740; *p *= .004; 95%CI = .602-.910) and approximately 10% less likely to be diagnosed with *depression *(OR = 0.881; p = .044; 95%CI = .780-.996) than those who had not grown up in the same rural community in which they were born. Similarly, individuals who had migrated between rural communities were approximately 50% less likely to be diagnosed with *adjustment reaction *(OR = 0.571; *p *< .001; 95%CI = .441-.739) than participants who stayed in the rural communities in which they were born. Interestingly, *nondependent drug abuse *was not significantly associated with rural stability (OR = 0.935; *p *> .05; 95%CI = .627-1.39) or migration between rural communities (OR = 1.42; *p *> .05; 95%CI = .952-2.11).

## Discussion

The present findings show that growing up in a rural environment or migrating between rural communities may protect against some mental health conditions, namely, acute reaction to stress, adjustment reaction, and depression. More specifically, youth and adults who grew up in the same rural community were at lower risk of being diagnosed with depression and adjustment reaction than individuals who did not grow up in the same rural community in which they were born, and children migrating between rural communities were at lower risk of being diagnosed with acute reaction to stress than participants who did not migrate between rural communities.

However, it is worth noting that for other mental health diagnoses we did not find a link with migration patterns. For example, we did not find significant differences between rural and urban environments or migration patterns between these two types of environments in the diagnosis of neurotic disorder, personality disorder, alcohol dependence, and nondependent drug abuse. Therefore we conclude that if rurality plays a protective role in the development of mental health, it does so only for specific conditions.

We argue that clues to what might be protecting children living in rural communities from developing acute reactions to stress, adjustment reaction, or depression may be suggested by a null finding. We found that, after controlling for important paternal sociodemographic characteristics, adolescents and young adults living in rural places are as likely to become nondependent drug abusers as individuals growing up in urban communities. We qualify this null finding as important because it is indeed consistent with our interpretation of the protective role of rurality. However, it is contrary to a literature showing that leisure boredom is associated with substance abuse [e.g., [28,39-41]], especially among rural youth [42-46], and suggesting that there may be some characteristics of rurality that put youth at risk for drug abuse.

It has been speculated that some of the alleged risk factors of rurality may be linked to the remoteness, isolation, and seclusion that generally are embedded in rural living and attract rural youth to large cities. Paradoxically, these features may relate to a perceived sense of status quo and lack of change. The underlying rationale is that the association between boredom and drug use in adolescents and young adults might be stronger in rural than in urban communities because living in rural communities might make individuals within these developmental periods more prone to boredom and, by implication, might make them experience less change or novelty than their counterparts living in urban communities.

Our analyses clearly show that, when individuals are matched for a series of family and socioeconomic variables, differences relative to nondependent drug abuse among rural and urban groups disappear. Thus, we conclude that it is possible that the differences found in relation to nondependent drug abuse reported in the literature may be due to the fact that the latent variable *boredom *may be confounded with uncontrolled socioeconomic and family variables, which instead reflect the typically greater availability of resources and access to services, facilities, and amenities enjoyed by urban populations.

Indeed, the pattern of results in our study suggests an alternative interpretation of the influence of rurality. That is, keeping constant the extent of access and resources varying with living contexts, rurality may well play a protective role for mental health of adolescents and young adults because it provides them with a needed sense of stability and control.

This proposed interpretation evokes a host of interesting questions concerning what is the optimal level of "social environment stimulation" in critical periods such as adolescence and young adulthood. Research addressing such questions has almost exclusively focused on infancy and early childhood, but seems to have largely neglected other developmental periods in the life span, and to have underestimated the role played by the context in which individuals live. Clearly, given the potential important links with lifestyle, well-being, and health outcomes, this should be an area of priority for future research.

While approaches to health policy tend to treat rural areas as uniform entities, mental health differences between rural areas may be as pronounced as those observed between urban and rural communities. There are an array of different and specific dimensions of rurality and urbanity that health researchers need to consider to better understand what community aspects may be associated with mental health outcomes [47]. For example, resource-dependent rural communities can be extremely different from one another, because farming, mining, and forestry are each affected differently by shifts in the market economy and availability of resources. Such shifts may also be partially responsible for individual trends in migration, which in turn represent an important element of the community social fabric. At the same time, the influences of rurality cannot be studied without controlling for individual-level characteristics that contribute to the socioeconomic profile of an entire community.

The present study has highlighted the important role played by stability, as opposed to migration, in contributing to the mental health of members of rural and urban communities. Our findings also suggest that important family characteristics such as sociodemographics, duration of employment, and a history of mental health may be possible confounders in previous studies in which differences between rural and urban communities have been identified.

While in this study we treated paternal characteristics (e.g., mental health diagnosis, work history, and ethnicity) as control variables, it is worth noting that these were consistently associated with increased risk of mental health diagnosis among the children. More specifically, paternal mental health diagnosis and Caucasian origins (compared to Chinese and Sikh) were associated with greater odds of mental health diagnosis among the children. These findings may explain in part some of the inconsistencies between rural and urban communities in drug use reported in the literature, as the inconsistent results could be confounded by factors such as ethnicity and familial history of mental health.

There are a number of limitations to this study that are worth mentioning. First, because our outcome measures were derived from medical records, we were not able to address the link between mental health and urbanity-rurality that may exist at the subclinical level, nor could we explore the role of potentially important contextual factors (e.g., social capital). Second, while we controlled for important sociodemographic and mental health characteristics of the fathers, we did not have access to maternal characteristics and therefore could not include them in this study. Third, the participants in the study represent a very specific population - that is, the children of male sawmill workers in British Columbia, Canada - and therefore findings from this study cannot be generalized. Finally, rural health researchers may be critical of our definition of rurality, which was solely based on population size (centers with less than 100,000 people), and our classification of migration patterns is reductive in that it did not divide urban migrators into those who migrated to other urban places and those who migrated from urban to rural places.

Because of the limitations of this study, further research on this topic needs to be conducted before recommendations for clinical practice can be extrapolated. Nonetheless, it is reasonable to advocate for a clinical practice that takes into consideration not only the individual histories of patients, but also the influence that broader social environments exert on the etiology of mental health conditions. This is a critical concept since it may have implications for treatment of the individual, but also for the identification of large-scale public mental health prevention programs.

## Conclusions

Thanks to the use of a relatively homogeneous sample, this study provides some compelling evidence of the protective role of rural environments in the development of *some *mental health conditions (i.e., depression, adjustment reaction, and acute reaction to stress) but not others (e.g., nondependent drug abuse).

## Abbreviations

BC: British Columbia; BCLHDB: British Columbia Linked Health Database; ICD: International Classification of Disease; OR: Odds Ratio; CI: 95% Confidence Interval.

## Competing interests

The authors declare that they have no competing interests.

## Authors' contributions

SM directed the analysis, and was the lead writer. AO was PI for purposes of obtaining funding for this research, and reviewed drafts. KC assisted with the literature review. RH conducted the analysis, and LC managed the database. AD contributed conceptually and reviewed drafts. CH conducted the research, helped direct the analysis, and read drafts of the paper. All authors read and approved the final manuscript.
